# Cancer during Adolescence: Negative and Positive Consequences Reported Three and Four Years after Diagnosis

**DOI:** 10.1371/journal.pone.0029001

**Published:** 2011-12-14

**Authors:** Gunn Engvall, Martin Cernvall, Gunnel Larsson, Louise von Essen, Elisabet Mattsson

**Affiliations:** 1 Department of Women's and Children's Health, Uppsala University, Uppsala, Sweden; 2 Department of Public Health and Caring Sciences, Psychosocial Oncology and Supportive Care, Uppsala University, Uppsala, Sweden; The University of Queensland, Australia

## Abstract

Persons diagnosed with cancer during adolescence have reported negative and positive cancer-related consequences two years after diagnosis. The overall aim was to longitudinally describe negative and positive cancer-related consequences reported by the same persons three and four years after diagnosis. A secondary aim was to explore whether reports of using vs. not using certain coping strategies shortly after diagnosis are related to reporting or not reporting certain consequences four years after diagnosis. Thirty-two participants answered questions about coping strategies shortly after diagnosis and negative and positive consequences three and four years after diagnosis. Answers about consequences were analysed with content analysis, potential relations between coping strategies and consequences were analysed by Fisher's exact test. The great majority reported negative and positive consequences three and four years after diagnosis and the findings indicate stability over time with regard to perceived consequences during the extended phase of survival. Findings reveal a potential relation between seeking information shortly after diagnosis and reporting a more positive view of life four years after diagnosis and not using fighting spirit shortly after diagnosis and not reporting good self-esteem and good relations four years after diagnosis. It is concluded that concomitant negative and positive cancer-related consequences appear stable over time in the extended phase of survival and that dialectical forces of negative and positive as well as distress and growth often go hand-in-hand after a trauma such as cancer during adolescence.

## Introduction

Adolescence is a time of great change that involves establishing identity and self-image, becoming autonomous from parents, and physical changes [1]. A cancer diagnosis during this time may affect the transition from childhood to adulthood and improved survival rates have resulted in more attention towards the psychological significance of cancer during adolescence [2].

While some studies conclude that cancer during adolescence has a severe negative psychosocial impact [3]–[5], other studies conclude that there are positive outcomes [6]–[11]. Prior findings show that persons diagnosed with cancer during adolescence, on a group level, report a steady increase in psychosocial function during the acute and extended phase of survival [6], [11]. Four years after diagnosis, they report a higher level of vitality and lower levels of anxiety and depression than a reference group consisting of healthy individuals in comparable ages [6]. Individuals exposed to a traumatic event such as cancer during adolescence may experience an acceleration of maturation, enhanced emotional development, and a heightened appreciation of life [7]–[10]. When considering these findings it should be taken into account that other findings show that some persons diagnosed with cancer during adolescence are characterised by psychosocial dysfunction still eighteen months after diagnosis [12].

It was not until the 1980 s that research focused on the possibility that suffering may cause a positive life change [13]. Descriptions of growth have been reported by people who have faced traumatic events [14]–[20] and include changes in self-perception, interpersonal relationships, and philosophy of life [13]. These changes have been conceptualised as coping [21]–[22] and as a transformation of the individual's understanding of the world [17], [23] i.e. posttraumatic growth [13]. It has been put forward that growth does not exclude distress and that manageable distress supports growth [13]. Findings from the paediatric oncology context support this reasoning [24]–[26] and show that greater perceived treatment severity [25], life threat [25], and intensity from cancer-related symptoms [24], [26] as well as symptoms of posttraumatic stress [25] are associated with growth among survivors of childhood cancer.

Prior findings show that persons diagnosed with cancer during adolescence report negative as well as positive cancer-related consequences two years after diagnosis [10]. The aims of the present study were to, for individuals diagnosed with cancer during adolescence: describe negative and positive cancer-related consequences reported three and four years after diagnosis; examine whether similar and/or different consequences are reported three and four years after diagnosis as two years after diagnosis, and explore if reports of using vs. not using certain coping strategies shortly after diagnosis are related to reporting or not reporting certain consequences four years after diagnosis. The findings can be of theoretical relevance for further research on psychosocial consequences of cancer during adolescence and may help to guide clinicians in their psychosocial work within paediatric oncology care.

## Methods

The study is part of a project focusing on short- and long-term psychosocial outcomes of cancer during adolescence to which 61 adolescents were recruited. Data have been/are collected from these persons at 4–8 weeks after diagnosis (T1), and at 6 (T2), 12 (T3), and 18 months (T4), and 2 (T5), 3 (T6), 4 (T7), and 10 years (T8) after diagnosis. Data collected at T1 and T5-T7 is presented in this report.

### Participants

Adolescents (13–19 years) diagnosed with cancer or a recurrence of cancer between 1999 and 2003 were recruited consecutively from three of the six Swedish paediatric oncology centres. To be eligible, the adolescent had to be Swedish speaking, diagnosed with cancer for the first time or with a recurrence of cancer after having been disease-free and off treatment for at least one year, treated with chemotherapy, and cognitively, emotionally, and physically able to participate. A co-ordinating nurse at each centre was responsible for recruitment and assessed, in collaboration with a treating physician, each adolescent's ability to participate.

During the time of recruitment 90 adolescents were diagnosed with cancer for the first time, whereas ten were diagnosed with a recurrence. Of these, 11 were not eligible due to the inclusion criteria. Of the eligible adolescents, 65 (73%) agreed to participate, of which four were excluded: two became too ill before the interview at T1, and two were missed due to administrative reasons. Hence, 61 (69%) adolescents were included, 56 newly diagnosed and five diagnosed with a recurrence. Reasons for attrition up to four years after diagnosis are: death (15 participants) and withdrawal (5). Nine persons did not participate at all assessments (9).

Data from the 32 participants (18 males and 14 females) who participated at T1 and T5–T7 are presented. At T1 30 were newly diagnosed whereas two were diagnosed with a recurrence. The participants were diagnosed with CNS-tumour (1 participant), Ewing sarcoma (1), Leukaemia (10), Lymphoma (13), Osteosarcoma (4), and other solid tumours (3). The mean age at T1 was 15.9 (sd.1.6) years. All, except one, were off treatment at T5, T6, and T7. One was diagnosed with a recurrence at T5 and T6 respectively.

### Ethics statement and data collection

Ethical approval was obtained from the local ethics committee at the faculty of medicine at the respective centre. A co-ordinating nurse at each centre provided potential participants and their parents, face-to-face, with oral and written information about the study such as the purpose of the research, approximately three weeks after diagnosis. A few days later, the adolescent was asked, face-to-face, about oral consent by the same nurse. If the adolescent was younger than 18 years, parents were asked to provide consent on behalf of the adolescent. Shortly before each data collection a co-ordinating nurse at the respective centre was contacted to ensure that the adolescent was still cognitively, emotionally, and physically able to participate. Due to long distances data were collected through telephone interviews from the Department of Public Health and Caring Sciences at Uppsala University. Interviews were performed by the last author who already at the time of data collection had extensive experience of interviewing patients. Our impression is that most respondents appreciated the relative anonymity of this procedure. This as well as the fact that they when asked about positive and negative consequences at T5–T7 previously had been interviewed four to six times within the same project may have increased their willingness to openly describe their experiences. At each interview participants were informed about the purpose of the research.

At T1 participants were asked structured questions regarding the extent to which he/she had used the coping strategies: accepting, distracting, fighting spirit, minimising, seeking information, and seeking support to cope with the following areas of distress: feelings of alienation (5 aspects), personal changes (5), physical concerns (5), and worries (5) [27]. The questions were answered on a six-point scale ranging from not at all to very much (coded 0–5), referring to the time since diagnosis. The choice of strategies was based on the available literature at the time [28]–[30], clinical experience of the members in the research group, and findings from pilot interviews with five healthy adolescents.

At T5–T7 participants were asked semi-structured questions about negative and positive cancer-related consequences. The questions were pilot-tested before posed to the participants. The interviewer was supportive and asked follow-up questions in order to help the respondent to elucidate his/her answers e.g. “Can you please elaborate on what you mean” or “Can you give an example of that”. The respondents were asked to answer according to their present situation. The answers lasted from a few minutes up to twenty minutes, were audiotape-recorded and transcribed verbatim.

### Data analysis

Answers to semi-structured questions about negative and positive cancer-related consequences were analysed by content analysis [31]–[32]. The manifest content i.e. what the text said, is presented in categories [31]. All authors read the transcribed text. Words and sentences (recording units) containing information regarding the questions were identified by the first, third and last author. Recording units were grouped into mutually exclusive categories by the first, third and last author, units in the same category are assumed to have a similar meaning. The first, third, and last author defined the boundaries of each category and the descriptions of the central characteristics of each category. If the content in a category identified at T6 and/or T7 corresponded with the content of a category identified at T5 [10], it was given the same name as that category. However, data collected at T6 and T7 were not analysed with the purpose to fit into the categories identified at T5. Even if a respondent mentioned a certain unit several times (at the same assessment), it was counted once in the result.

The Statistical Package for the Social Sciences (SPSS) version 17.0 was used to, by means of Fisher's exact test, explore potential relations between reports of using vs. not using certain coping strategies at T1 and reporting or not reporting certain consequences at T7. A participant's use of a certain strategy was calculated as the sum (range 0–20) of the scores reported for that strategy divided by the number of areas (range 0–4) for which the strategy was reported. Thus, the mean value for a certain strategy varies between 0 and 5. In this report a strategy is defined as 'used' by a participant if its mean value was 2.6 or higher and 'not used' when its mean value was lower.

The study has in most part been reported according to the COREQ checklist, see Text S1.

## Results

Identified categories of cancer-related consequences, category content, and examples of statements in each category three and four years after diagnosis are presented in Table S1.

The number of persons reporting only negative, negative and positive, or only positive consequences at two (T5), three (T6), and four (T7) years after diagnosis are presented in Table S2. The majority described negative and positive consequences at all assessments. Only one person at T5 and T6 and two persons at T7 reported only negative consequences whereas seven persons at T5 and three persons at T6 and T7 reported only positive consequences. One person did not report any negative or positive consequence at T6 and T7.

Two categories not identified at T5 [10] were identified, negative self-esteem at T6 and time consumption and financial issues at T6 and T7.

See Table S3 for a presentation of reports of using vs. not using certain coping strategies at T1 in relation to reporting or not reporting certain consequences at T7. Findings reveal a potential relation between seeking information shortly after diagnosis and reporting a more positive view of life four years after diagnosis and not using fighting spirit shortly after diagnosis and not reporting good self-esteem and good relations four years after diagnosis.

## Discussion

This longitudinal research indicates the existence of a basic human protective system when struck with cancer during adolescence. Three and four years after diagnosis most participants describe negative as well as positive cancer-related consequences within physical, emotional, social, cognitive, and financial domains. The consequences are almost the same as those reported two years after diagnosis [10]. The findings indicate potential relationships between using vs. not using certain coping strategies shortly after diagnosis and experiencing vs. not experiencing certain consequences four years after diagnosis.

Positive changes in the perception of self, in the relationships with others, and in the general philosophy of life were revealed, supporting previous findings [9], [33]–[35]. It is reasonable to assume that cancer during adolescence is challenging enough to set in motion the cognitive processes hypothesised to lead to a positive psychological change i.e. posttraumatic growth [13]. The findings indicate, as put forward by others, that distress does not exclude growth and that experiences of feeling more vulnerable yet stronger may co-exist [36]. The following statement illustrates this: “I worry about having a relapse. It's always on my mind and I'm always afraid. However, despite the worries I see life from a new angle. Actually, I've grown as a person and my self-confidence is much better. I believe in myself in another way now”. Some participants reported symptoms of posttraumatic stress e.g. flash backs and nightmares about the cancer. Previous findings have revealed a curvilinear relation between posttraumatic growth and symptoms of posttraumatic stress, with the strongest relation between growth and a moderate level of symptoms [37]. Such a relation could partly explain mixed findings [38] with regard to the relation between distress and growth, others have put forward that distress and growth are independent dimensions [39]. The distinction between the assumptions is important as it has different implications on how to alleviate distress, the latter suggesting that interventions aiming at alleviating distress not necessarily facilitate growth [38].

In order to identify adolescents who experience cancer-related psychosocial dysfunction and thus need extra psychological care or treatment assessments of distress as well as coping strategies should be made during the acute and extended phase of survival. These assessments could help to sort responses in a clinically meaningful way [40]. Psychological interventions should include problem solving strategies, imaginable exposure methods, and cognitive reappraisal. Acceptance-based interventions [41] balancing acceptance and change to help individuals to act effectively in accordance with personal values in the presence of interfering thoughts, emotions, and bodily sensations could be a viable option. Whether diminishing distress encourages growth could be investigated in trials investigating the clinical efficacy of psychological interventions to alleviate emotional distress experienced by adolescents with cancer.

The participants' descriptions of positive consequences during the extended phase of survival correspond with previous findings illustrating that their psychosocial function change for the better over time. A majority have reported good, or even excellent, psychosocial function eighteen months after diagnosis [12] and a higher level of vitality and lower levels of anxiety and depression than a healthy reference group in comparable age four years after diagnosis and on a group-level [6]. Several factors have been identified as protective when struck by adversity, for example good cognitive abilities and self-esteem [42]. Close relationships with parents, other adults and peers, parents' education and socioeconomic status, effective schools and good public health care with a high availability are other protective factors [43]. In this study it was investigated whether using vs. not using certain coping strategies shortly after diagnosis is related to experiences of distress as well as growth four years after diagnosis. The findings indicate a potential relation between seeking information shortly after diagnosis and reporting a more positive view of life four years after diagnosis and not using fighting spirit shortly after diagnosis and not reporting good self-esteem and good relations four years after diagnosis. When considering these findings it should be taken into account that they are based on data from few individuals and that a relatively large number of analysis were performed to analyse potential relations between coping strategies and consequences.

It has been put forward that individuals who do not construe positive consequences from a trauma differ in cognitive processing variables, coping, personality characteristics, and/or social support from those who construe benefits [44]. Future research should try to reveal the importance of cognitive processing, as well as close relationships, on responses to cancer during adolescence. It could be speculated that the person/s who only reported negative consequences were medically worse off e.g. diagnosed with a recurrence, than the others. This speculation partly holds true as one of these persons was diagnosed with a recurrence. However the other three persons who were diagnosed with a recurrence reported negative as well as positive consequences. Three individuals included in the project died between three and four years after diagnosis and are thus not included in the sample of the present study. It could be speculated that these persons would not report any positive consequences. However two of these persons reported positive as well as negative consequences three years after diagnosis, i.e. at the last assessment before they died.

The findings show that most participants report negative and positive cancer-related consequences during the extended phase of survival and indicate stability over time with regard to perceived consequences. We consider the findings worthwhile to consider in themselves as well as to formulate hypotheses for future research. It should however be considered that the results are based on self-reports and alternative hypotheses to distress and growth such as time, denial, defensiveness, social desirability, and impression management [45]–[47] should be tested in future research. Future research should also try to reveal the perceived intensity of the consequences identified in this research. The findings build on categorical data and conclusions regarding the extent to which the negative as well as positive consequences are experienced cannot be drawn. Future research should also try to reveal whether distress and growth after a trauma such as cancer during adolescence are dependent or independent phenomena. The findings indicate a relationship between using vs. not using certain coping strategies shortly after diagnosis and experiencing certain consequences four years after diagnosis. In spite of the relatively limited sample size and the way coping was measured and analyzed we believe that the findings are interesting enough to generate ideas for future research in which sufficiently large samples to reach adequate power to identify relationships where they exist should be used. This poses a challenge to the psychosocial paediatric-oncology research community due to the low cancer incidence among adolescents and international efforts may be necessary to reach this end.

### Conclusion

The findings show concomitant negative and positive cancer-related consequences during the extended phase of survival and that dialectical forces of negative and positive, distress and growth often go hand-in-hand after a trauma such as cancer during adolescence.

## Supporting Information

Table S1 **A presentation of identified categories, category content, and examples of statements about negative and positive cancer-related consequences three (T6) and four (T7) years after diagnosis (N = 32).**
(DOC)

Table S2 **A presentation of the number of persons reporting only negative, negative and positive, and only positive cancer-related consequences two^a^ (T5), three^b^ (T6), and four^b^ (T7) years after diagnosis.**
(DOC)

Table S3 **A presentation of the number of participants^a^ reporting using and not using a certain coping strategy shortly after diagnosis (T1) and reporting and not reporting a certain consequences four years after diagnosis (T7) (N = 32).**
(DOC)

Text S1 **The COREQ checklist completed for the study.**
(DOC)
