# Supplementary material for: Cancer during Adolescence: Negative and Positive Consequences Reported Three and Four Years after Diagnosis
Source: PLoS One. 2011 Dec 14;6(12):e29001. doi: 10.1371/journal.pone.0029001 (PMC3237575; doi:10.1371/journal.pone.0029001)
Supplement: Table S1 — A presentation of identified categories, category content, and examples of statements about negative and positive cancer-related consequences three (T6) and four (T7) years after diagnosis (N = 32). (DOC) [file pone.0029001.s001.doc]

Table S1. A presentation of identified categories, category content, and examples of statements about negative and positive cancer-related consequences three (T6) and four (T7) years after diagnosis (N=32).

| **Categories** | **Category content** | **Examples of statements** |
| --- | --- | --- |
| ***Negative consequences*** |  |  |
| Bodily concerns | Physical and appearance problems, and the consequences of these. | *“One of my legs hurts more or less the whole time…it’s my knee that hurts quite a bit when I walk, so I can’t run or anything like that because I’ve got a droopy foot…so when it comes to sports, activities like that are not so cool.”* (Respondent 27, male, 23 years) |
| Unpleasant thoughts and feelings | Unpleasant thoughts and feelings, e.g. with regard to memories from the hospital visits, and in connection with questions about the disease. “Feeling blue”, depression and sorrow over lost years. | *“Maybe it’s now that I have sort of begun to understand that I’ve been ill*... *I dream nightmares every night about all kinds of things and then I sleep very badly as well and that if anything is a sign that you’re worried or something like that… it feels as if I lost two of my teenage years like, they’re just gone and you can’t do anything about it.”* (Respondent 13, female, 17 years) |
| Outside the circle of friends | Being isolated or having lost touch with friends. | “*I think that other people who have not experienced the same thing seem to find it difficult to understand how it has been, like. I think that they don’t understand how bad you can feel, it seems like that.”* (Respondent 28, female, 17 years) |
| Difficulties with school-work/work | Schoolwork takes a lot of effort and energy and extra work is necessary to catch up on missed time at school. Missing days at work. | *“Last winter I missed some lectures and examinations… and now I don’t have enough results for this semester.”* (Respondent 6, male, 19 years) |
| Negative self-esteem | Increased shyness and insecurity, more withdrawn, which negatively affects self-confidence and self-image. | “*I’ve become shy. I’ve become this withdrawn and shy because I think that people can see that I’m different.”* (Respondent 25, female, 20 years) |
| Time consumption and financial issues | Extra time, expense, and missing study grants due to disease and follow-up. | *“It takes a lot of time, it sucks, it’s the journey...”* (Respondent 17, male, 16 years) |
| ***Positive consequences*** |  |  |
| A more positive view of life | Another view of life. An awareness of death means that problems assume other proportions and that the present and day-to-day life are at the centre of things. | *“You appreciate slightly different things that people do not usually think about, like how it smells at home…sitting and having a cup of tea, you can get this feeling that this is really good.”* (Respondent 25, female, 20 years) |
| Good relations | Good ability to understand and, therefore, help other people. Values close relations with family members, friends, and other people. | *“I talk more about my feelings now...and therefore I have actually become more close to my schoolmates...and of course very close to mom...".”* (Respondent 5, female, 16 years) |
| Good self-esteem | An inner change with regard to maturity and development into a responsible person with good self-esteem, among other things regarding the person’s own body. | *“I think I’ve grown as a person, actually. It feels as if I now have more self-confidence and yes, you believe in yourself in another way*.” (Respondent 4, male, 20 years) |
| Knowledge and experience with regard to disease and hospital care | Knowledge and experience of disease and hospital care and a secure relationship with hospital care. | *“Well it’s an experience to have been ill… it’s something that I carry with me…”* (Respondent 1, female, 19 years) |
| Broader perspectives | New leisure activities and occupational plans. | *“I have got some new interests… billiard. It’s fun to compete with someone else, so to say*.” (Respondent 9, male, 20 years) |
| Material gains | New things, social insurance etc. | *“…Well some material benefits… when I became healthy I got a horse*…” (Respondent 18, female, 16 years) |
