# Supplementary material for: Cancer during Adolescence: Negative and Positive Consequences Reported Three and Four Years after Diagnosis
Source: PLoS One. 2011 Dec 14;6(12):e29001. doi: 10.1371/journal.pone.0029001 (PMC3237575; doi:10.1371/journal.pone.0029001)
Supplement: Table S2 — A presentation of the number of persons reporting only negative, negative and positive, and only positive cancer-related consequences twoa (T5), threeb (T6), and fourb (T7) years after diagnosis. (DOC) [file pone.0029001.s002.doc]

Table S2. A presentation of the number of persons reporting only negative, negative and positive, and only positive cancer-related consequences two^a^ (T5), three^b^ (T6), and four^b^ (T7) years after diagnosis.

|  | **Only negative** | | | **Negative and positive** | | | **Only positive** | | |
| --- | --- | --- | --- | --- | --- | --- | --- | --- | --- |
|  | T5 n=1 | T6 n=1 | T7 n=2 | T5 n=24 | T6 n=27 | T7 n=26 | T5 n=7 | T6 n=3 | T7 n=3 |
| ***Negative consequences*** |  |  |  |  |  |  |  |  |  |
| Bodily concerns | 1 | 1 | 2 | 18 | 20 | 18 |  |  |  |
| Unpleasant thoughts and feelings | - | - | - | 13 | 10 | 7 |  |  |  |
| Outside the circle of friends | - | - | - | 5 | 4 | 8 |  |  |  |
| Difficulties with school-work/work | - | - | 1 | 4 | 1 | 4 |  |  |  |
| Negative self-esteem | - | - | - | - | 4 | - |  |  |  |
| Time consumption and financial issues | - | - | - | - | 3 | 1 |  |  |  |
| ***Positive consequence*** |  |  |  |  |  |  |  |  |  |
| A more positive view of life |  |  |  | 14 | 17 | 17 | 2 | 3 | 2 |
| Good relations |  |  |  | 10 | 14 | 11 | 1 | 1 | 2 |
| Good self-esteem |  |  |  | 9 | 12 | 14 | 3 | 1 | 1 |
| Knowledge and experience with regard to disease and hospital care |  |  |  | 11 | 10 | 10 | 2 | - | 1 |
| Broader perspectives |  |  |  | 4 | 3 | 2 | - | - | - |
| Material gains |  |  |  | 2 | 3 | 3 | - | 1 | - |

^a^N=32, ^b^N=31
