# Supplementary material for: Cancer during Adolescence: Negative and Positive Consequences Reported Three and Four Years after Diagnosis
Source: PLoS One. 2011 Dec 14;6(12):e29001. doi: 10.1371/journal.pone.0029001 (PMC3237575; doi:10.1371/journal.pone.0029001)
Supplement: Table S3 — A presentation of the number of participantsa reporting using and not using a certain coping strategy shortly after diagnosis (T1) and reporting and not reporting a certain consequences four years after diagnosis (T7) (N = 32). (DOC) [file pone.0029001.s003.doc]

Table S3. A presentation of the number of participants^a^ reporting using and not using a certain coping strategy shortly after diagnosis (T1) and reporting and
not reporting a certain consequences four years after diagnosis (T7) (N=32).

| **Coping strategies** | | **Negative consequences** | | | | **Positive consequences** | | | | | | | | | | | |
| --- | --- | --- | --- | --- | --- | --- | --- | --- | --- | --- | --- | --- | --- | --- | --- | --- | --- |
|  | | Bodily concerns | | | | A more positive view of life | | | | Good self-esteem | | | | Good relations | | | |
|  |  | Reported (n=20) | | Not reported (n=12) | | Reported (n=19) | | Not reported (n=13) | | Reported (n=15) | | Not reported (n=17) | | Reported (n=13) | | Not reported (n=19) | |
|  | (n) | n | (%) | n | (%) | n | (%) | n | (%) | n | (%) | n | (%) | n | (%) | n | (%) |
| Accepting | Used (28) | 18 | (64) | 10 | (36) | 16 | (57) | 12 | (43) | 12 | (43) | 16 | (57) | 13 | (46) | 15 | (54) |
|  | Not used (4) | 2 | na | 2 | na | 3 | na | 1 | na | 3 | na | 1 | na | 0 | na | 4 | na |
| Distracting | Used (16) | 11 | (69) | 5 | (31) | 8 | (50) | 8 | (50) | 6 | (38) | 10 | (63) | 8 | (50) | 8 | (50) |
|  | Not used (16) | 9 | (56) | 7 | (44) | 11 | (69) | 5 | (31) | 9 | (56) | 7 | (44) | 5 | (31) | 11 | (69) |
| Fighting spirit | Used (17) | 10 | (59) | 7 | (41) | 12 | (71) | 5 | (29) | 11 | (65) | 6 | (35) | 10 | (59) | 7 | (41) |
|  | Not used (15) | 10 | (67) | 5 | (33) | 7 | (47) | 8 | (53) | 4 | na | 11* | (73) | 3 | na | 12* | (80) |
| Minimising | Used (20) | 12 | (60) | 8 | (40) | 12 | (60) | 8 | (40) | 10 | (50) | 10 | (50) | 10 | (50) | 10 | (50) |
|  | Not used (12) | 8 | (67) | 4 | na | 7 | (58) | 5 | (42) | 5 | (42) | 7 | (58) | 3 | na | 9 | (75) |
| Seeking information | Used (20) | 12 | (60) | 8 | (40) | 15* | (75) | 5 | (25) | 9 | (45) | 11 | (55) | 9 | (45) | 11 | (55) |
|  | Not used (12) | 8 | (67) | 4 | na | 4 | na | 8 | (67) | 6 | (50) | 6 | (50) | 4 | na | 8 | (67) |
| Seeking support | Used (17) | 9 | (53) | 8 | (47) | 10 | (59) | 7 | (41) | 9 | (53) | 8 | (47) | 7 | (41) | 10 | (59) |
|  | Not used (15) | 11 | (73) | 4 | na | 9 | (60) | 6 | (40) | 6 | (40) | 9 | (60) | 6 | (40) | 9 | (60) |

^a^ Percentages are calculated when n > 5

*p < .05
