# Supplementary material for: Cancer during Adolescence: Negative and Positive Consequences Reported Three and Four Years after Diagnosis
Source: PLoS One. 2011 Dec 14;6(12):e29001. doi: 10.1371/journal.pone.0029001 (PMC3237575; doi:10.1371/journal.pone.0029001)
Supplement: Text S1 — The COREQ checklist completed for the study. (DOC) [file pone.0029001.s004.doc]

The COREQ checklist by Tong, Sainsbury and Craig (2007) completed for the study: Cancer during adolescence: negative and positive consequences reported three and four years after diagnosis.

| *Domain 1: Research team and reflexivity* |
| --- |
| Personal characteristics |
| 1. It is reported that the last author conducted the interviews. |
| 1. Credentials are reported, when performing the study the third, fourth, and last author had PhD exams whereas the first and second authors were PhD students. By now the first author has passed her PhD examination. |
| 1. The authors’ occupations are not reported. When writing the paper the first and second authors were PhD students, the third author was occupied as a university lecturer, the fourth author as a professor and the last author as a researcher. By now the first author is a university lecturer, the second author a PhD student, the third author a university lecturer, the fourth author a professor and the fifth author a researcher. |
| 1. It is reported (by first names) that the second author is male and that the first, third, fourth, and last authors are females. |
| 1. The third, fourth, and last authors have extensive training and experience in performing qualitative (and quantitative) research. The first and second authors were PhD students by the time of the study and still have relatively limited experience of research. However, all authors have extensive experience in interviewing patients. The first, third, and last authors are RNs whereas the second and fourth authors are clinical psychologists. It is mentioned that the interviewer, i.e. the last author already at the time of data collection had extensive experience of interviewing. |
| Relationship with participants |
| 1. It is reported that at the first assessment (4-8 weeks after diagnosis) the participants had had no prior contact with the interviewers/researchers. At the second, third and fourth assessment the participants had previously been interviewed and thus in contact with the interviewers/researchers four to six times previously. |
| 1. It is reported that all participants were informed both at the time of information and at all interviews about the purpose of the research. |
| 1. Interviewer characteristics were not reported to participants. |
|  |
| *Domain 2: Study design* |
| Theoretical framework |
| 1. It is reported that data about cancer-related consequences were analyzed with content analysis. |
| Participant selection |
| 1. It is reported that participants were included consecutively. |
| 1. It is reported that potential participants were approached face-to-face when provided with information about the study and when asked about consent. It is also reported that all interviews were conducted via telephone. |
| 1. It is reported that the sample consists of 32 individuals. |
| 1. The number of eligible participants, the number who agreed to participate and the attrition rate is reported. |
| Setting |
| 1. It is reported that data were collected via telephone from the Department of Public Health and Caring Sciences, Uppsala University. We assume that most participants were in their homes when interviewed, they can however have been somewhere else when interviewed via mobile phones. These aspects are not mentioned. |
| 1. Nobody besides the interviewer was present at the Department of Public Health and Caring Sciences during the interviews. We do not know whether someone was present around the participants when interviewed. However, our impression is that this was not so. This is not mentioned. |
| 1. Important characteristics of the sample are reported. |
| Data collection |
| 1. It is reported that interview questions were pilot-tested and that the interviewer was supportive and asked follow-up questions in order to help the respondent to elucidate his/her answers. |
| 1. It is reported that participants were asked the same questions about cancer-related consequences at two, three, and four years after diagnosis. It is also mentioned that this circumstance may have affected their willingness to answer the questions. |
| 1. It is reported that the interviews were audio-taped. |
| 1. Field notes were not taken as the interviews were conducted via telephone. This is not mentioned. |
| 1. It is reported that the interviews about cancer-related consequences lasted between a few minutes up to twenty minutes. |
| 1. Data saturation is not discussed. |
| 1. Transcripts were not returned to participants. This is not mentioned. |
| *Domain 3: Analysis and findings* |
| Data analysis |
| 1. It is reported that the first, third, and last author coded the data. |
| 1. The coding tree, i.e. the steps by which interview data were coded, is reported. |
| 1. It is reported that the themes were derived from data. |
| 1. No software was used to handle data. This is not mentioned. |
| 1. Participants did not provide feedback on findings. This is not mentioned. |
| Reporting |
| 1. Participants’ quotations are presented to illustrate categories and for each quotation the respondent’s number is reported. |
| 1. There is consistency between the data presented and the findings. |
| 1. The themes negative and positive consequences and the categories belonging to these themes are clearly presented. |
| 1. There is a description of diverse cases: of participants only reporting negative and positive consequences respectively and all categories derived from interview data no matter the number of respondents mentioning an answer referred to a certain category are presented. |
